# Supplementary material for: Estimation of joint torque in dynamic activities using wearable A-mode ultrasound
Source: Nat Commun. 2024 Jul 9;15:5756. doi: 10.1038/s41467-024-50038-0 (PMC11233567; doi:10.1038/s41467-024-50038-0)
Supplement: Supplementary file 1 — Supplementary Information [file 41467_2024_50038_MOESM1_ESM.pdf]

SUPPLEMENTARY INFORMATION

Estimation of Joint Torque in Dynamic Activities Using Wearable A-Mode Ultrasound

Yichu Jin, Jonathan T. Alvarez, Elizabeth L. Suitor, Krithika Swaminathan, Andrew Chin, Umut S. Civici, Richard W. Nuckols, Robert D. Howe, Conor J. Walsh

SUPPLEMENTARY METHODS

Assessing A-mode echo intensity using a mechanical testing system

We conducted an experiment using a mechanical testing system (Instron, USA) to quantify the relationship between A-mode echo intensity and the angle of incidence. In this experiment, we mounted a SET on the mechanical tester and cyclically displaced it towards and away from a 3D printed reflector (Fig. S2a). The mechanical tester was programmed to oscillate three times for 10 mm at three different frequencies (0.5, 0.75, and 1 Hz). We placed the reflector inside a jar of water with the SET remaining in contact with the water throughout the test. Water was used as the test medium because its speed of sound and acoustic impedance approximated the acoustic properties of muscle tissue. This experiment was repeated with seven reflectors, each designed with a different boundary angle, including 0°, 2.5°, 5°, 7.5°, 10°, 12.5°, and 15°. For each reflector, we calculated the mean and SD of peak echo intensity across all time frames including all speeds and repetitions. The 0° boundary angle (90° angle of incidence) generated the strongest echoes, and the mean peak echo intensity dropped drastically with increasing boundary angle, with approximately 95% signal loss from 0° to 5°, as shown in Fig. S2b.

Evaluating the MBTA with simulated data

To evaluate the performance of the MBTA, we artificially modified the data from the mechanical test. We multiplied the raw ultrasound data from the 5° reflector with a sinusoidal scaling signal between 1 and 20 (Fig. S3a). We made this modification to simulate the effect of changing boundary angle, specifically ranging between 0° and 5°. We used the displacement measurements from the mechanical tester as the ground truth and applied the MBTA to the simulated data. MBTA generated accurate displacement tracking, whereas the brightness-based and the cross-correlation-based methods exhibited high-frequency noise and low-frequency drifts, respectively (Fig. S3b). Quantitatively, the MBTA achieved an RMSE of 0.05 mm and NRMSE of 0.5%, which were lower than either the brightness-based method (RMSE = 0.19 mm, NRMSE = 1.9%) or the cross-correlation-based method (RMSE = 0.73 mm, NRMSE = 7.3%) alone.

Comparing muscle thickness tracking using A-mode and B-mode ultrasound

We conducted an in vivo experiment to evaluate the utility of A-mode ultrasound for tracking changes in muscle thickness. We simultaneously collected A-mode and B-mode ultrasound of the quadriceps while a participant performed five repetitions of concentric and eccentric knee extensions at 90° s^{-1} on a dynamometer (Fig. S4a). We designed a 3D printed case that held an A-mode SET and a B-mode linear array transducer (LAT; MicroUs, Telemed, Lithuania) positioned 2 cm apart (Fig. S4b). This transducer assembly was placed over the RF muscle belly and secured to the leg using self-adhesive wraps (Coban Wrap, 3M, USA). To synchronize these ultrasound signals, we recorded sync pulses from both ultrasound systems (A-mode: 360 Hz; B-mode: 80 Hz) using the DAQ unit. In B-mode processing, we first cropped ultrasound images to isolate the target muscle boundaries. We then found the 2D muscle boundary from each B-mode image by identifying a line spanning across the full width of the image and produced the highest mean pixel intensity (Fig. S4c). Lastly, we extracted a single depth value from the identified line at the location corresponding to SET placement, recorded it over time, and used the resulting time-series data as the ground truth muscle thickness (Fig. S4d). In A-mode processing, we estimated the depth of the same muscle boundary using the MBTA (Fig. S4e). In this experiment, we measured the depth of the deep boundary of the vastus intermedius muscle (VI) relative to the skin, thereby capturing the combined thickness change of both the RF and the VI. Comparing to the B-mode

measurements, A-mode ultrasound achieved accurate muscle thickness tracking during controlled dynamic contractions with an RMSE of 0.48 mm and a NRMSE of 1.7%.

Sensitivity analysis on transducer placement

We conducted a single-participant sensitivity analysis to understand the effect of transducer placement. Specifically, we drew a 3 (in the lateral-medial direction with resolution of 10 mm) by 6 (in the distal-proximal direction with resolution of 35 mm) grid on the participant's quadriceps above the RF muscle. We then placed the SETs on each location within the grid and asked the participant to perform four repetitions of passive, concentric, and eccentric knee extensions at 60° s^{-1} on the dynamometer. This process was repeated for each of the 18 locations. We processed the ultrasound and dynamometer data using the same procedures as in the dynamometer test. At each location, we evaluated the ultrasound signal quality as well as the fitted $(MT, Ang)^2$ model's correlation to ground truth knee torque (Fig. S9).

Knee torque estimation during stationary cycling

We conducted a single-participant study comparing knee torque estimation from the RF and the VL during stationary biking. For each muscle, the participant conducted a dynamometer calibration and a cycling validation. During calibration, the participant performed five repetitions of passive, concentric, and eccentric contractions of knee extensors at 90° s^{-1} on the dynamometer. During validation, the participant performed 30 seconds of stationary biking on a cycle ergometer (AtomX, Wattbike, UK) at a constant rate of 60 RPM under low resistance (80 W), medium resistance (150 W), and high resistance (250 W). SETs were placed on the right leg over either the RF or VL muscle belly, and two wireless IMUs were attached to the lateral sides of the right thigh and shank (Fig. S10a). Knee torque was measured from the dynamometer during calibration, and knee angle was tracked using IMUs during both calibration and validation. In post-processing, all data were low pass filtered at 5 Hz. We used the calibration data to fit $(MT, Ang)^2$ models for correlating muscle thickness (either RF or VL) and IMU-based knee angle to dynamometer-based knee torque. These fits were then applied to the validation data for knee torque estimation during biking. We only analyzed the data from the middle 20 cycling cycles for each condition to minimize the potential impact of inconsistent cycling rates at the beginning and end of the trial. The estimated torques were segmented based on crank angle using an external IMU attached to the pedal for visualization (Fig. S10b). Notably, the RF and VL tests were conducted on two separate days. As a result, the difference in knee angles from the two tests (Fig. S10c, Fig. S10d) can likely be attributed to variations in IMU placement.

SUPPLEMENTARY FIGURES

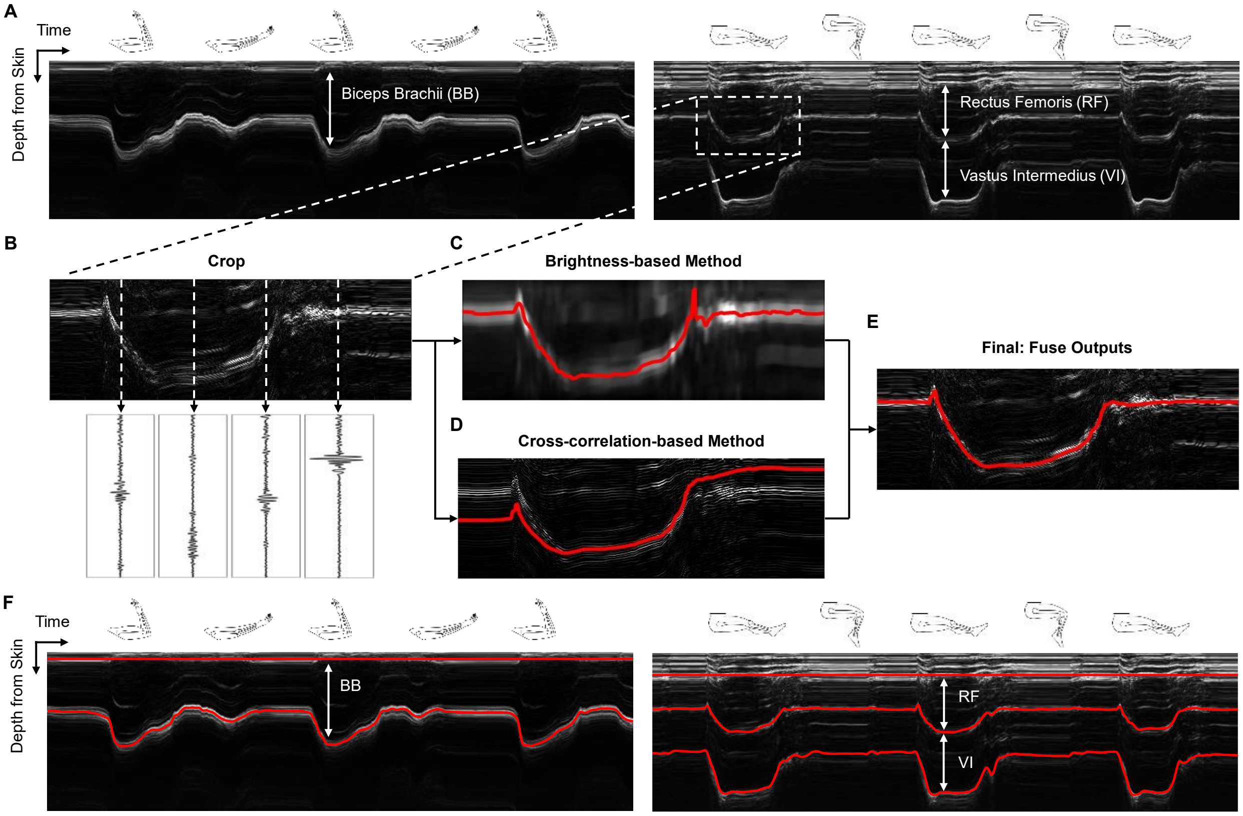

Supplementary Figure 1: Overview of the muscle boundary tracking algorithm (MBTA). (**A**) Representative A-mode ultrasound data during concentric elbow flexions (left) and knee extensions (right). (**B**) Manually cropped A-mode data to focus on the muscle boundary of interest (deep RF boundary in example). Insets show raw ultrasound data at different time frames. (**C**) Tracing (red line) from the brightness-based method overlaid on top of smoothed A-mode data. (**D**) Tracing (red line) from the cross-correlation-based method overlaid on top of raw A-mode data. (**E**) Final tracing from the MBTA which fuses the outputs from both methods for robustness against high-frequency noises (from the brightness-based method) and low-frequency drifts (from the cross-correlation-based method). (**F**) MBTA-traced boundaries of the biceps brachii (BB), rectus femoris (RF), and vastus intermedius (VI) (red lines) overlaid on A-mode ultrasound data from (A).

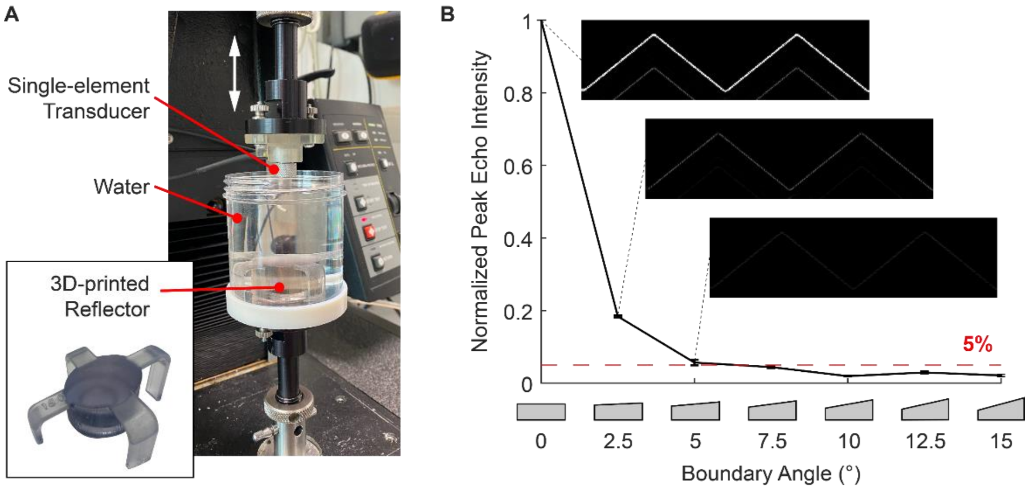

Supplementary Figure 2: A-mode echo intensity assessment. (**A**) Photograph of the experimental setup with a zoom-in view of the 3D-printed reflector (bottom left). (**B**) Mean normalized peak echo intensity decreased with boundary angle. The error bar represents the SD (*n* = 576 samples). Insets show representative A-mode ultrasound data of reflectors with different boundary angles. Red dashed line represents 5% of the peak echo intensity observed at a 0° boundary angle.

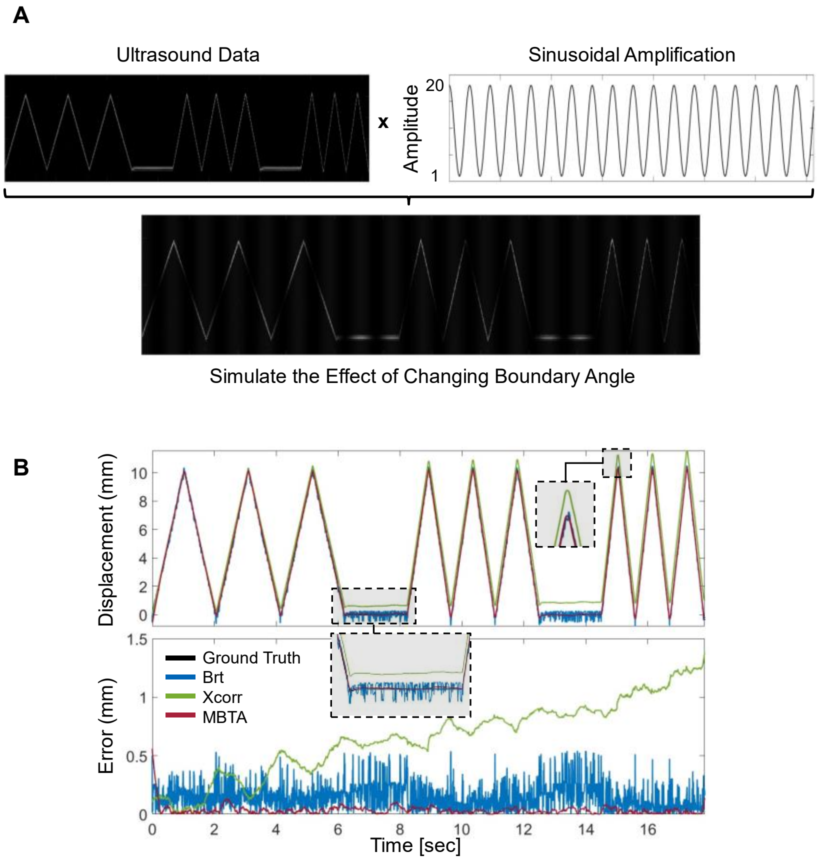

Supplementary Figure 3: Evaluation of the MBTA using simulated data. (**A**) Raw ultrasound data from the 5° reflector are artificially amplified with a sinusoidal wave signal to simulate the effect of changes in angle of incidence. (**B**) Displacement estimates and errors from the brightness-based (Brt), cross-correlation-based (Xcorr), and the final MBTA method. Displacement measurements from the mechanical testing system are used as the ground truth.

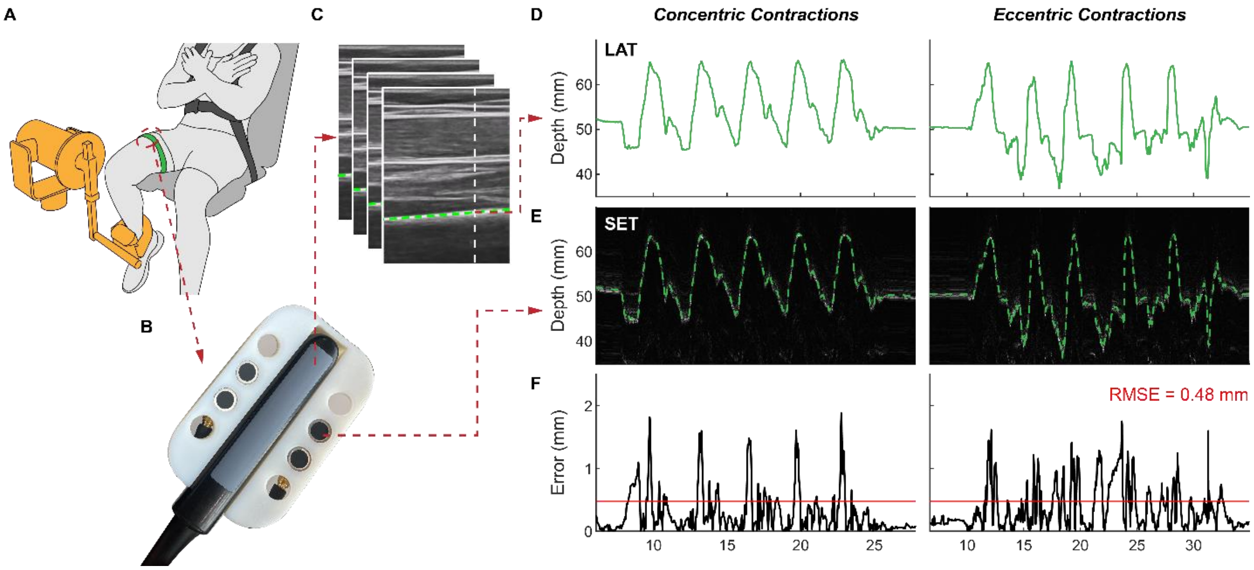

Supplementary Figure 4: Comparison of A-mode and B-mode ultrasound for muscle thickness tracking. (**A**) Illustration of the experimental setup. (**B**) Photograph of the 3D printed case that can mount up to eight A-mode SETs and a B-mode linear array transducer (LAT). (**C**) Identification of 2D muscle boundary (green dashed line) using the B-mode image at each time frame. (**D**) B-mode measured time-series muscle thickness plots obtained by extracting depth values from the identified muscle boundaries near the SET placement [white vertical dashed line in (C)]. (**E**) A-mode ultrasound data with MBTA tracing (green dashed line) overlaid. (**F**) Time-series error plots comparing A-mode-based with B-mode-based muscle thickness measurements. Red lines represent the RMSE of 0.48 mm.

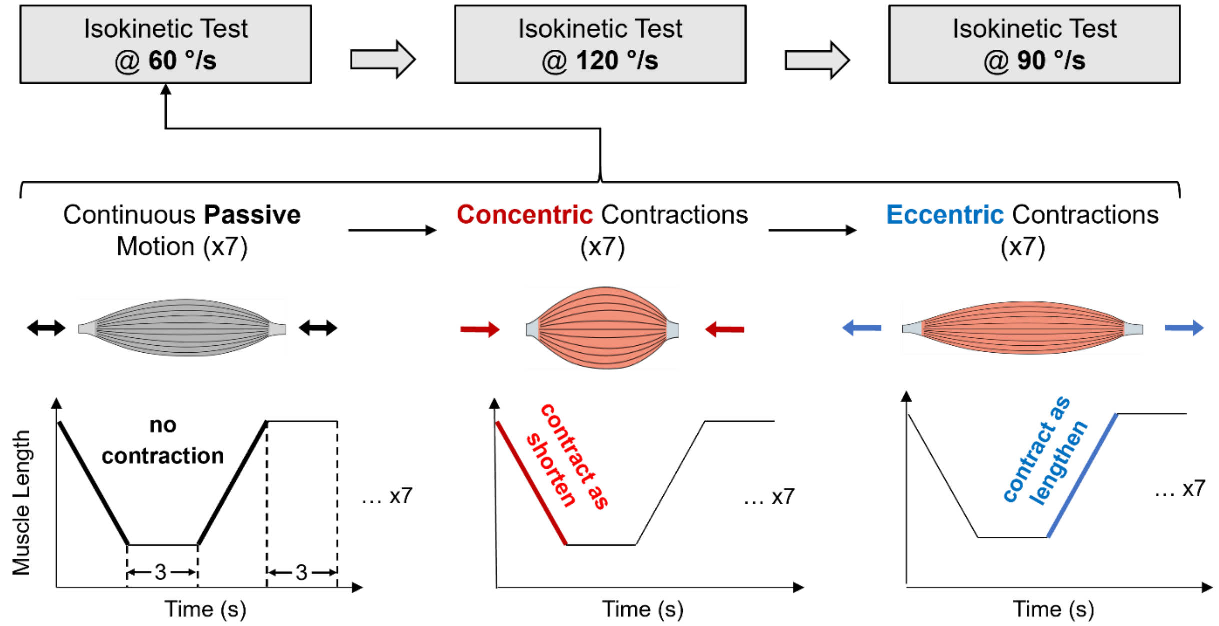

Supplementary Figure 5: Experimental protocol for isokinetic dynamometer tests.

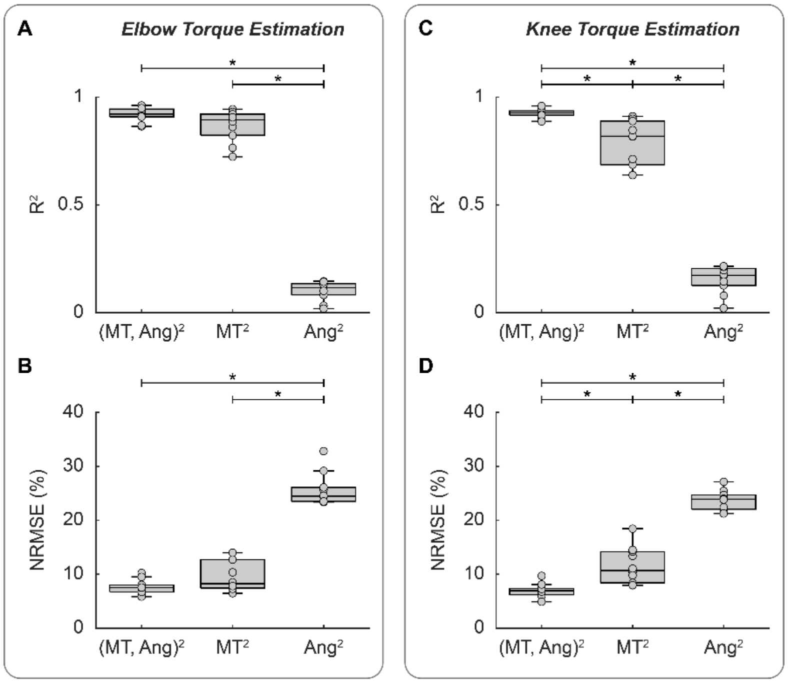

Supplementary Figure 6: Evaluation of input contributions in dynamometer tests. (**A**) R^2 values for different input variables across all participants (*n* = 10) from the elbow test. One-way ANOVA: significant main effects ($p < 0.001$, $F_{2,18} = 1001$). Bonferroni post-hoc analysis: (MT, Ang)^2 vs MT^2 ($p = 0.090$), (MT, Ang)^2 vs Ang^2 ($p < 0.001$), and MT^2 vs Ang^2 ($p < 0.001$). (**B**) NRMSEs for different input variables across all participants (*n* = 10) from the elbow test. Friedman's test: significant main effects ($p < 0.001$, $\chi^2_2 = 20$). Bonferroni post-hoc analysis: (MT, Ang)^2 vs MT^2 ($p = 0.312$), (MT, Ang)^2 vs Ang^2 ($p < 0.001$), and MT^2 vs Ang^2 ($p < 0.001$). (**C**) R^2 values for different input variables across all participants (*n* = 10) from the knee test. One-way ANOVA: significant main effects ($p < 0.001$, $F_{2,18} = 434$). Bonferroni post-hoc analysis: (MT, Ang)^2 vs MT^2 ($p = 0.003$), (MT, Ang)^2 vs Ang^2 ($p < 0.001$), and MT^2 vs Ang^2 ($p < 0.001$). (**D**) NRMSEs for different input variables across all participants (*n* = 10) from the knee test. One-way ANOVA: significant main effects ($p < 0.001$, $F_{2,18} = 275$). Bonferroni post-hoc analysis: (MT, Ang)^2 vs MT^2 ($p < 0.001$), (MT, Ang)^2 vs Ang^2 ($p < 0.001$), and MT^2 vs Ang^2 ($p < 0.001$). For (A) – (D), each box bounds the interquartile range (IQR) divided by the median with whiskers extending up to 1.5*IQR. Gray dots represent the metric value for each participant. *$p < 0.05$. (MT, Ang)^2 represents quadratic fits with both muscle thickness and joint angle as input variables. MT^2 represents quadratic fits with only muscle thickness as the input variable. Ang^2 represents quadratic fits with only joint angle as the input variable.

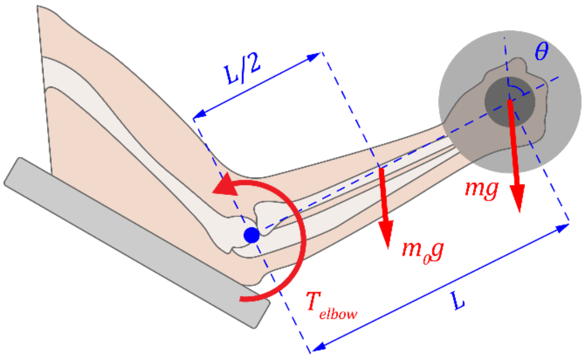

Supplementary Figure 7: Free body diagram of the dumbbell curl.

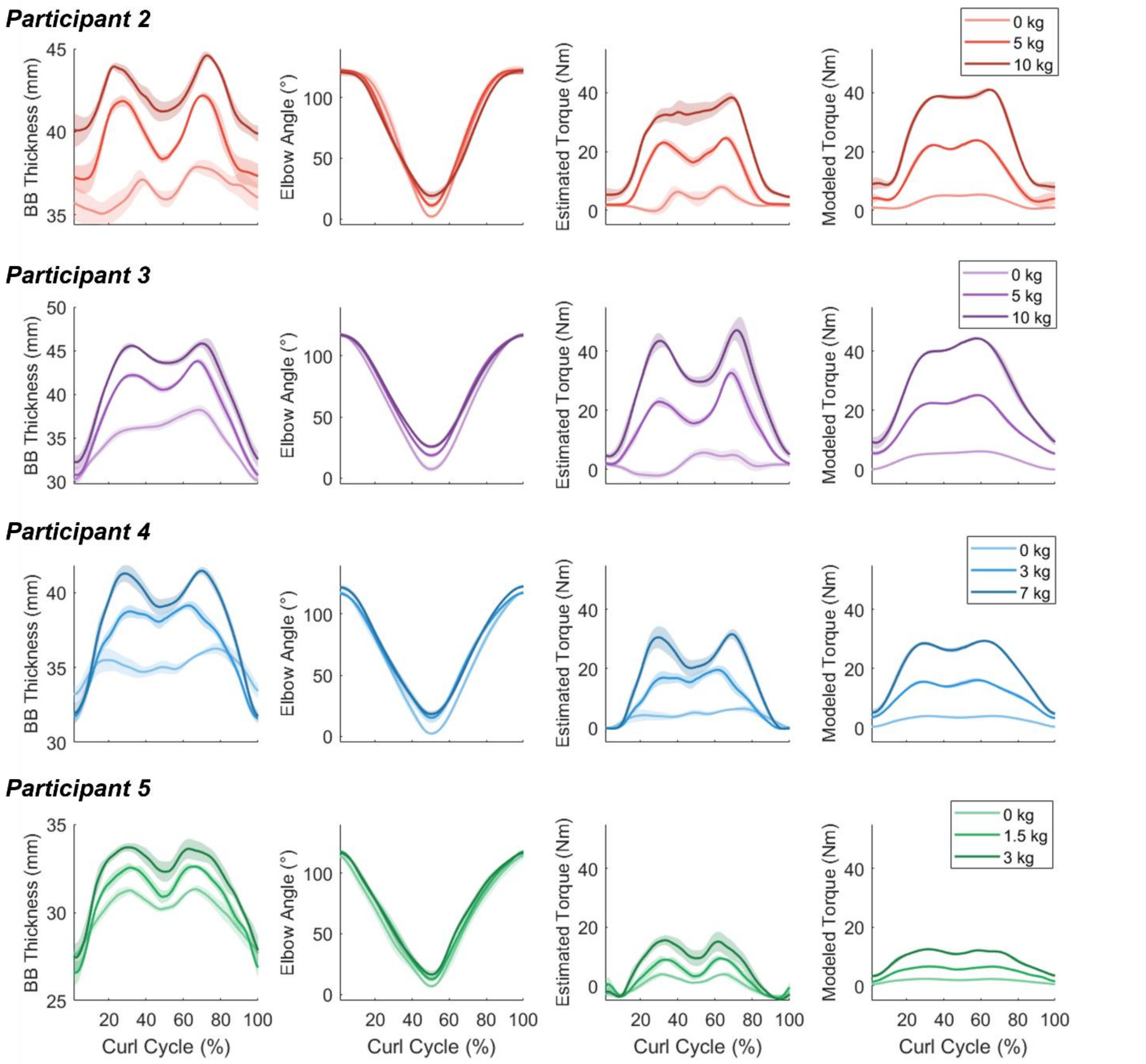

Supplementary Figure 8: Data from different participants during the dumbbell curl study. Each row represents BB thickness (left column), elbow angle (center left column), estimated elbow torque (center right column), and calculated elbow torque from a rigid body model (right column) for one participant during curls with different dumbbell weights. Data from Participant 1 is shown in Fig. 3. Lines and shaded regions represent mean ± SD (n = 6 repetitions).

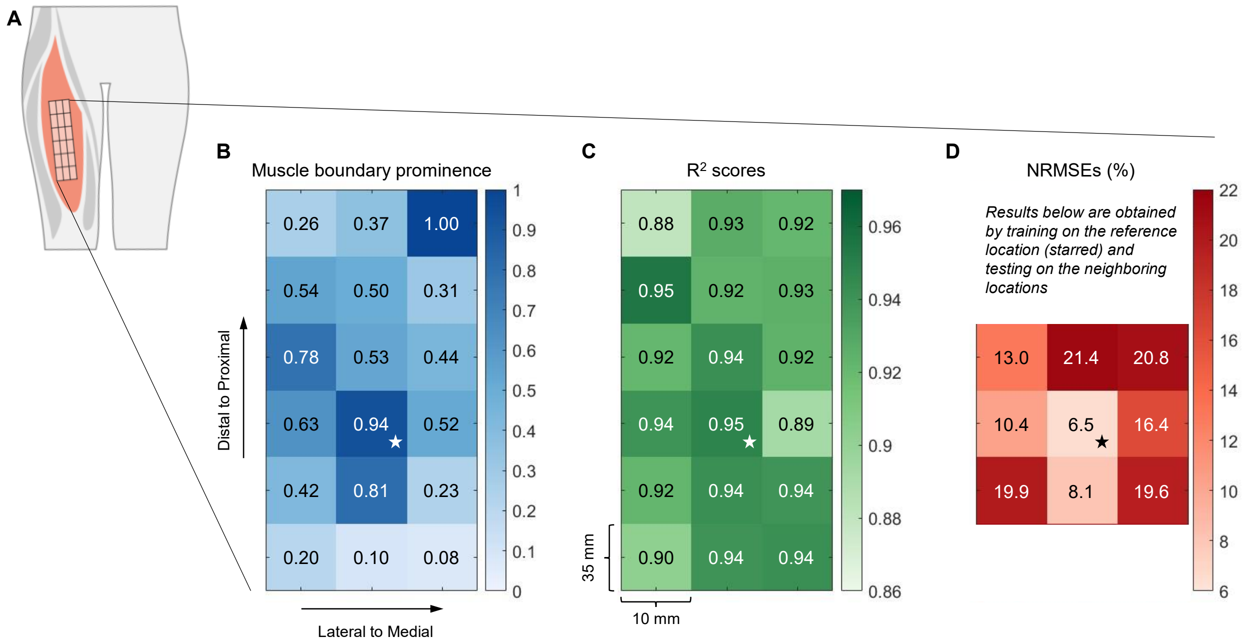

Supplementary Figure 9: Sensitivity analysis on transducer placement. (**A**) SETs were placed on 18 different locations (3 by 6 grid) across the RF muscle. At each location, the participant performed 4 repetitions of passive, concentric, and eccentric knee extensions on the dynamometer. (**B**) Muscle boundary prominence scores from all tested locations. This score is designed to quantitatively assess the ultrasound signal quality of the deep RF muscle boundary. Specifically, this score is calculated by averaging the maximum peak prominence of the raw ultrasound data within the region of interest across all times frames and then normalizing the scores from all locations. (**C**) Coefficients of determination scores for $(MT, Ang)^2$ models from all locations. Each model was obtained using the ultrasound and dynamometer data collected at the respective location. (**D**) Normalized RMSEs obtained by fitting a $(MT, Ang)^2$ model on the reference location [starred in (B), (C), and (D)] and evaluated on the 8 neighboring locations.

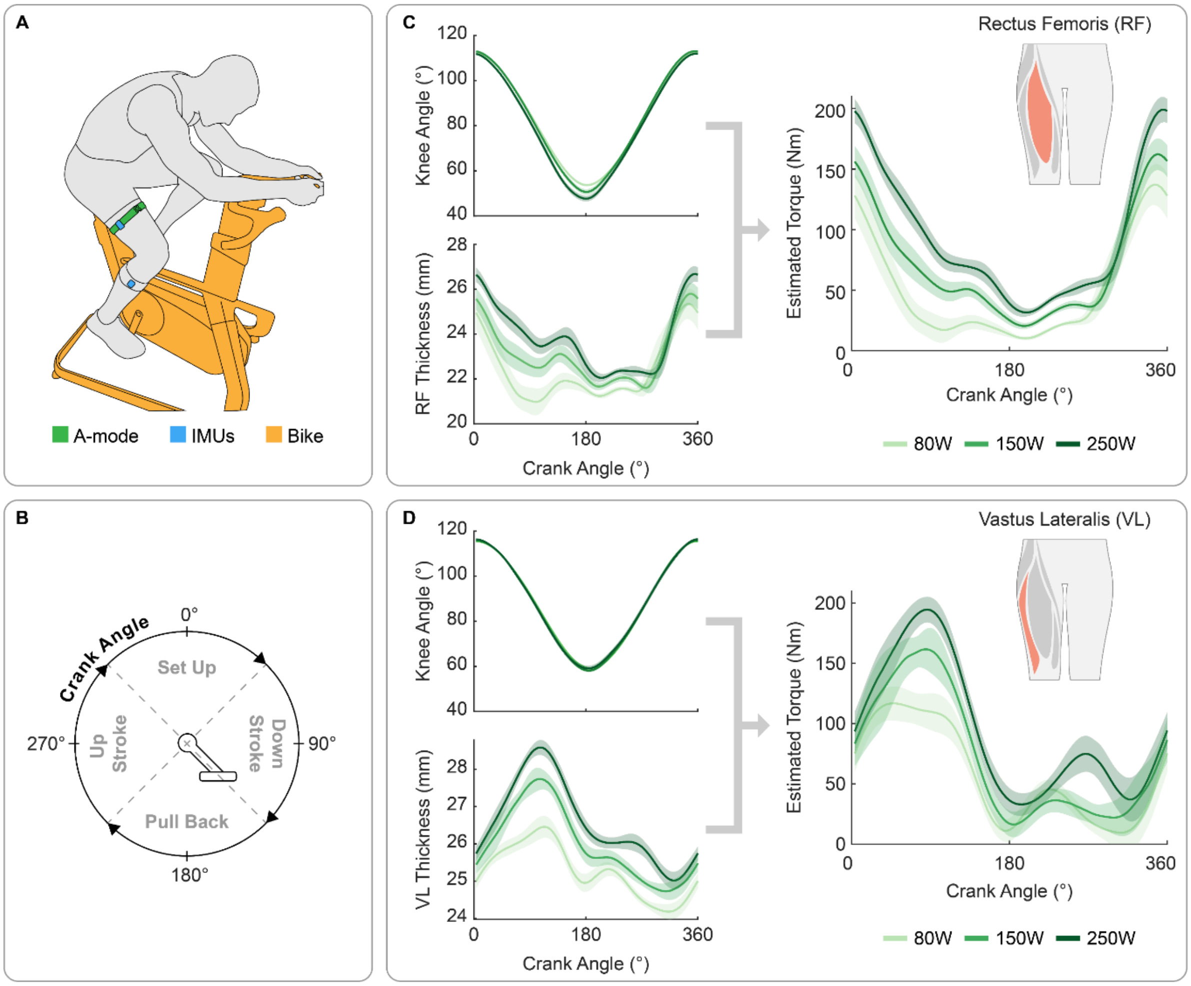

Supplementary Figure 10: Knee torque estimation during cycling. (**A**) Illustration of a single participant cycling on a stationary bike with the RF or VL muscle thickness measured with SETs and knee angle measured with wireless IMUs. (**B**) Illustration of crank angle and stroke phase definition. (**C**) Knee angle (top left), RF thickness (bottom left), and estimated knee torque (right) during cycling at low (80W), medium (150W), and high (250W) resistance. Lines and shaded regions represent mean ± SD (*n* = 20 cycling cycles). (**D**) Knee angle (top left), VL thickness (bottom left), and estimated knee torque (right) during cycling at different resistance. Lines and shaded regions represent mean ± SD (*n* = 20 cycling cycles).

SUPPLEMENTARY TABLES

Supplementary Table 1: Estimation accuracies for (MT, Ang)^2 models in isokinetic dynamometer tests.

	Elbow			Knee		
	R^2	RMSE (Nm)	NRMSE (%)	R^2	RMSE (Nm)	NRMSE (%)
Individualized Models						
Participant 1	0.91	2.83	7.6	0.93	10.95	6.2
Participant 2	0.86	4.22	10.2	0.92	12.76	6.8
Participant 3	0.96	3.56	6.2	0.94	15.77	7.1
Participant 4	0.93	4.47	6.7	0.94	12.62	5.9
Participant 5	0.87	3.45	9.5	0.93	8.78	6.8
Participant 6	0.94	3.39	5.8	0.92	12.97	7.4
Participant 7	0.93	3.40	6.7	0.96	9.57	4.9
Participant 8	0.91	5.67	7.5	0.90	19.23	9.7
Participant 9	0.91	4.74	8.0	0.92	13.24	7.3
Participant 10	0.95	3.20	7.5	0.89	10.03	8.1
Mean ± SD	0.92 ± 0.03	3.89 ± 0.86	7.6 ± 1.4	0.92 ± 0.02	12.59 ± 3.12	7.0 ± 1.3
Generalized Models						
All Participants	0.82	N/A	11.5	0.78	N/A	12.0

Generalized models were obtained using normalized muscle thickness and joint torque data (normalization to account for individual differences in muscle size and strength). Therefore, RMSEs for generalized models are not available.

Supplementary Table 2: Estimation accuracies in dumbbell curl and treadmill locomotion tests.

	Calibration for dumbbell curls			Treadmill locomotion		
	R^2	RMSE (Nm)	NRMSE (%)	R^2	RMSE (Nm)	NRMSE (%)
Participant 1	0.96	2.69	7.3	0.93	21.94	5.3
Participant 2	0.97	2.48	5.1	0.94	14.82	5.0
Participant 3	0.95	3.92	8.1	0.89	22.76	7.3
Participant 4	0.97	1.97	5.4	0.88	17.12	7.2
Participant 5	0.94	2.04	7.9	0.94	10.50	5.5
Mean ± SD	0.96 ± 0.02	2.62 ± 0.78	6.8 ± 1.4	0.92 ± 0.03	17.43 ± 5.09	6.0 ± 1.1

Supplementary Table 3: Participant information for dynamometer tests.

	Gender	Age	Height (cm)	Mass (kg)	Dominant side
Participant 1	F	26	162	58	Right
Participant 2	F	29	160	59	Right
Participant 3	M	29	183	83	Right
Participant 4	M	33	177	77	Right
Participant 5	F	28	164	52	Right
Participant 6	M	27	180	88	Left
Participant 7	M	37	184	77	Right
Participant 8	M	25	189	80	Right
Participant 9	M	30	181	70	Right
Participant 10	M	26	170	62	Right

Supplementary Table 4: Participant information for dumbbell curl and treadmill locomotion tests.

	Gender	Age	Height (cm)	Mass (kg)	Dominant side
Participant 1	M	29	183	83	Right
Participant 2	M	24	193	84	Left
Participant 3	M	27	180	88	Left
Participant 4	M	30	181	70	Right
Participant 5	F	29	160	59	Right
