# Supplementary material for: Estimation of joint torque in dynamic activities using wearable A-mode ultrasound
Source: Nat Commun. 2024 Jul 9;15:5756. doi: 10.1038/s41467-024-50038-0 (PMC11233567; doi:10.1038/s41467-024-50038-0)
Supplement: Supplementary file 3 — Reporting Summary [file 41467_2024_50038_MOESM3_ESM.pdf]

nature portfolio

Corresponding author(s): Conor J. Walsh

Last updated by author(s): Jun 15, 2024

# Reporting Summary

Nature Portfolio wishes to improve the reproducibility of the work that we publish. This form provides structure for consistency and transparency in reporting. For further information on Nature Portfolio policies, see our Editorial Policies and the Editorial Policy Checklist.

## Statistics

For all statistical analyses, confirm that the following items are present in the figure legend, table legend, main text, or Methods section.

| n/a | Confirmed | |
|---|---|---|
| ☐ | ☒ | The exact sample size ($n$) for each experimental group/condition, given as a discrete number and unit of measurement |
| ☐ | ☒ | A statement on whether measurements were taken from distinct samples or whether the same sample was measured repeatedly |
| ☐ | ☒ | The statistical test(s) used AND whether they are one- or two-sided<br>*Only common tests should be described solely by name; describe more complex techniques in the Methods section.* |
| ☒ | ☐ | A description of all covariates tested |
| ☐ | ☒ | A description of any assumptions or corrections, such as tests of normality and adjustment for multiple comparisons |
| ☐ | ☒ | A full description of the statistical parameters including central tendency (e.g. means) or other basic estimates (e.g. regression coefficient) AND variation (e.g. standard deviation) or associated estimates of uncertainty (e.g. confidence intervals) |
| ☐ | ☒ | For null hypothesis testing, the test statistic (e.g. $F$, $t$, $r$) with confidence intervals, effect sizes, degrees of freedom and $P$ value noted<br>*Give P values as exact values whenever suitable.* |
| ☒ | ☐ | For Bayesian analysis, information on the choice of priors and Markov chain Monte Carlo settings |
| ☒ | ☐ | For hierarchical and complex designs, identification of the appropriate level for tests and full reporting of outcomes |
| ☒ | ☐ | Estimates of effect sizes (e.g. Cohen's $d$, Pearson's $r$), indicating how they were calculated |

*Our web collection on statistics for biologists contains articles on many of the points above.*

## Software and code

Policy information about availability of computer code

| | |
|---|---|
| Data collection | A-mode ultrasound was collected using MATLAB (R2023b). Dynamometer data was collected using LabChart 8. IMU data was collected using Movella DOT App (2023.6.0). Motion capture and ground reaction force data were collected using Qualisys (2023). B-mode ultrasound data was collected using EchoWave II (4.2.0). Instron data was collected using Bluehill Universal. |
| Data analysis | Data analysis was performed using MATLAB (R2023b). |

For manuscripts utilizing custom algorithms or software that are central to the research but not yet described in published literature, software must be made available to editors and reviewers. We strongly encourage code deposition in a community repository (e.g. GitHub). See the Nature Portfolio guidelines for submitting code & software for further information.

## Data

Policy information about availability of data

All manuscripts must include a data availability statement. This statement should provide the following information, where applicable:

- Accession codes, unique identifiers, or web links for publicly available datasets
- A description of any restrictions on data availability
- For clinical datasets or third party data, please ensure that the statement adheres to our policy

All data supporting the findings of this study are available within the article and its supplementary files. Any additional requests for information can be directed to, and will be fulfilled by, the corresponding author. Source data are provided with this paper.

## Research involving human participants, their data, or biological material

Policy information about studies with human participants or human data. See also policy information about sex, gender (identity/presentation), and sexual orientation and race, ethnicity and racism.

| | |
|---|---|
| Reporting on sex and gender | In this study, we recruited a total of 11 participants (8 male and 3 female). Gender was not a factor in our experiment design. The gender information was provided by participants through self-reporting. Both sex and gender are not relevant to the experiments or results of this study. |
| Reporting on race, ethnicity, or other socially relevant groupings | All participants were recruited randomly. No information on race, ethnicity, or any socially relevant grouping is relevant to the experiments or results of this study. |
| Population characteristics | The participants in this study are Harvard-affiliated members, ranging in age from 24 to 37 years old. No specific population characteristic is relevant to the experiments or results of this study. |
| Recruitment | Participants were recruited through word of mouth within the Harvard community. |
| Ethics oversight | Harvard Medical School Institutional Review Board (IRB #22086) |

Note that full information on the approval of the study protocol must also be provided in the manuscript.

# Field-specific reporting

Please select the one below that is the best fit for your research. If you are not sure, read the appropriate sections before making your selection.

☒ Life sciences ☐ Behavioural & social sciences ☐ Ecological, evolutionary & environmental sciences

For a reference copy of the document with all sections, see nature.com/documents/nr-reporting-summary-flat.pdf

# Life sciences study design

All studies must disclose on these points even when the disclosure is negative.

| | |
|---|---|
| Sample size | We recruited 10 participants in the dynamometer test, 5 participants in the dumbbell curl and treadmill locomotion tests, and 1 participant in the cycling and outdoor locomotion tests. The chosen sample sizes of 5 and 10 are comparable to those in similar biological torque estimation studies, which typically range from 5 to 8 participants. We justify the single participant in the cycling and outdoor locomotion tests by the absence of ground truth in these tests and the primary objective of these tests, which is a functional demonstration rather than quantitative validation. |
| Data exclusions | No data was excluded from analysis. |
| Replication | We quantitatively validated our torque estimation method with multiple participants, each performing at least five contractions in each test condition. Our reported results, generated using data from all participants and contractions, demonstrate the reproducibility of our experimental findings. |
| Randomization | For each experiment, all participants underwent the same set of test conditions in non-randomized order. The order of these conditions is not relevant to the results of this study because sufficient rest was provided between conditions to prevent fatigue. |
| Blinding | The investigators were not blinded to group allocation during data collection and analysis. Blinding is not relevant to the experiments or results of this study. |

# Reporting for specific materials, systems and methods

We require information from authors about some types of materials, experimental systems and methods used in many studies. Here, indicate whether each material, system or method listed is relevant to your study. If you are not sure if a list item applies to your research, read the appropriate section before selecting a response.

## Materials & experimental systems

| n/a | Involved in the study |
| --- | --- |
| ☒ | ☐ Antibodies |
| ☒ | ☐ Eukaryotic cell lines |
| ☒ | ☐ Palaeontology and archaeology |
| ☒ | ☐ Animals and other organisms |
| ☒ | ☐ Clinical data |
| ☒ | ☐ Dual use research of concern |
| ☒ | ☐ Plants |

## Methods

| n/a | Involved in the study |
| --- | --- |
| ☒ | ☐ ChIP-seq |
| ☒ | ☐ Flow cytometry |
| ☒ | ☐ MRI-based neuroimaging |

# Plants

| | |
| --- | --- |
| Seed stocks | *Report on the source of all seed stocks or other plant material used. If applicable, state the seed stock centre and catalogue number. If plant specimens were collected from the field, describe the collection location, date and sampling procedures.* |
| Novel plant genotypes | *Describe the methods by which all novel plant genotypes were produced. This includes those generated by transgenic approaches, gene editing, chemical/radiation-based mutagenesis and hybridization. For transgenic lines, describe the transformation method, the number of independent lines analyzed and the generation upon which experiments were performed. For gene-edited lines, describe the editor used, the endogenous sequence targeted for editing, the targeting guide RNA sequence (if applicable) and how the editor was applied.* |
| Authentication | *Describe any authentication procedures for each seed stock used or novel genotype generated. Describe any experiments used to assess the effect of a mutation and, where applicable, how potential secondary effects (e.g. second site T-DNA insertions, mosiacism, off-target gene editing) were examined.* |
